# Deep-learning-driven dose prediction and verification for stereotactic radiosurgical treatment of isolated brain metastases

**DOI:** 10.3389/fonc.2023.1285555

**Published:** 2023-11-20

**Authors:** Jinghui Pan, Jinsheng Xiao, Changli Ruan, Qibin Song, Lei Shi, Fengjiao Zhuo, Hao Jiang, Xiangpan Li

**Affiliations:** ^1^ School of Electronic Information, Wuhan University, Wuhan, Hubei, China; ^2^ Department of Radiation Oncology, Renmin Hospital, Wuhan University, Wuhan, Hubei, China; ^3^ Department of Oncology, Renmin Hospital, Wuhan University, Wuhan, Hubei, China; ^4^ Department of Radiation Oncology, Jiangling County People’s Hospital, Jingzhou, Hubei, China

**Keywords:** brain metastases, stereotactic radiosurgery, dose prediction, deep learning, radiation oncology

## Abstract

**Purpose:**

While deep learning has shown promise for automated radiotherapy planning, its application to the specific scenario of stereotactic radiosurgery (SRS) for brain metastases using fixed-field intensity modulated radiation therapy (IMRT) on a linear accelerator remains limited. This work aimed to develop and verify a deep learning-guided automated planning protocol tailored for this scenario.

**Methods:**

We collected 70 SRS plans for solitary brain metastases, of which 36 cases were for training and 34 for testing. Test cases were derived from two distinct clinical institutions. The envisioned automated planning process comprised (1): clinical dose prediction facilitated by deep-learning algorithms (2); transformation of the forecasted dose into executable plans via voxel-centric dose emulation (3); validation of the envisaged plan employing a precise dosimeter in conjunction with a linear accelerator. Dose prediction paradigms were established by engineering and refining two three-dimensional UNet architectures (UNet and AttUNet). Input parameters encompassed computed tomography scans from clinical plans and demarcations of the focal point alongside organs at potential risk (OARs); the ensuing output manifested as a 3D dose matrix tailored for each case under scrutiny.

**Results:**

Dose estimations rendered by both models mirrored the manual plans and adhered to clinical stipulations. As projected by the dual models, the apex and average doses for OARs did not deviate appreciably from those delineated in the manual plan (P-value≥0.05). AttUNet showed promising results compared to the foundational UNet. Predicted doses showcased a pronounced dose gradient, with peak concentrations localized within the target vicinity. The executable plans conformed to clinical dosimetric benchmarks and aligned with their associated verification assessments (100% gamma approval rate at 3 mm/3%).

**Conclusion:**

This study demonstrates an automated planning technique for fixed-field IMRT-based SRS for brain metastases. The envisaged plans met clinical requirements, were reproducible across centers, and achievable in deliveries. This represents progress toward automated paradigms for this specific scenario.

## 1 Introduction

Brain metastases rank as the most ubiquitous intracranial tumors and significantly contribute to the elevated mortality and disability rates associated with cancer. An estimated 25-40% of malignant neoplasms culminate in brain metastases (1, 2). Such metastatic occurrences predominantly manifest in patients diagnosed with non-small cell lung, breast, and melanoma. However, they can also arise from gastrointestinal tract malignancies, liver, pancreas, uterus, ovary, thyroid, adrenal gland, prostate, kidney, and bone (3). Present-day therapeutic interventions primarily involve surgery and radiotherapy, with Stereotactic radiosurgery (SRS) and whole brain radiation therapy (WBRT) (4, 5) emerging as the leading radiotherapeutic techniques. Due to its spatial dose distribution, exemplary conformal shape, reduced treatment sessions, enhanced tumor control rate and diminished adverse effects on healthy tissues compared to WBRT, SRS often holds a therapeutic advantage. Consequently, SRS is extensively employed in treating brain metastases (6, 7).

Current treatment planning strategies generally use the inverse intensity-modulated radiotherapy (IMRT) method, in which a clinical goal is first determined, followed by optimization of dose distribution to meet this goal. Physicists generally need to adjust the optimization parameters repeatedly to obtain a clinically plausible plan due to factors such as dose limitations for organs at risk, multileaf collimator physical constraints, etc. (8, 9). This planning procedure is time-consuming, labor-intensive, and may result in inconsistent treatment quality. Thus, automatic planning methods have been proposed to improve the quality and efficiency of IMRT planning. For example, the knowledge-based automatic planning method (10–12) predicts the dose distribution for a new patient by training on the dataset of accepted clinical plans. The predicted plan provides a better starting point, thereby reducing the frequency of trial and error. However, this method has certain limitations. On one hand, the method extracts the hand-design features from the patient’s anatomy, which separates the processes of feature extraction and dose prediction, resulting in a suboptimal solution; on the other hand, the predicted dose is usually a one-dimensional dose-volume histogram (DVH) or zero-dimensional dose point parameter, which cannot accurately reflect the three-dimensional (3D) dose distribution.

In recent years, advanced deep learning methods have made great progress in the automatic segmentation of organs at risk and multimodal image registration. This success has also inspired research on end-to-end 3D dose prediction using deep learning. In light of the excellent performance of ResNet (13) in image classification, Chen (14) and Fan (15) proposed deep learning methods based on ResNet to predict the 3D dose distribution of IMRT plans for head and neck cancer. However, ResNet performs resolution reduction operations on the images, resulting in low resolution of the predicted doses. To overcome this problem, Nguyen (16) introduced the 3D UNet (17) network, originally used for image segmentation, to facilitate dose prediction during IMRT planning of prostate cancer. UNet (18, 19) has a unique resolution-preserving feature that improves the resolution of the predicted dose distributions. UNet-based dose prediction has been predominantly applied to non-stereotactic intensity modulated radiation therapy (IMRT) plans (20–24), with limited exploration for stereotactic radiation therapy. A few studies have utilized UNet for stereotactic body radiation therapy (SBRT) dose prediction, including Kearney et al. (25) for prostate cancer and Momin et al. (26) for pancreatic cancer. Zhang et al. (27) developed a UNet model to predict Gamma Knife radiosurgery dose distributions for intracranial tumors. However, the application of deep learning for dose prediction in the specific scenario of linear accelerator-based stereotactic radiosurgery using fixed-field IMRT for brain metastases has not been extensively studied. Moreover, some commercial vendors such as Brainlab and Varian have partially implemented automated planning products using deep learning, though these are not tailored for the particular scenario addressed here.

Furthermore, while deep learning for dose prediction has been widely reported, conversion and delivery verification of predicted doses into clinically viable treatment plans remains an unmet challenge. Dose distributions forecasted on a voxel basis may not be achievable in practice due to beam and collimator limitations.

In summary, automated treatment planning for brain metastasis radiosurgery is an active research area with multiple academic and commercial solutions in various stages of development. Groups such as Zhang et al. have explored techniques like deep learning for this application. Commercial products from vendors including Brainlab and Varian perform automated planning tasks for radiosurgery of brain metastases using diverse approaches including deep-learning. However, exploring novel approaches may offer opportunities to improve on existing techniques. Here we describe a deep learning approach to planning brain metastasis radiosurgery as delivered by a linac radiosurgery platform employing a co-planar fixed-field IMRT delivery technique.

We proposed an automated treatment planning pipeline that integrates dose prediction, plan generation, and delivery verification. Two 3D UNet architectures were implemented to forecast dose distributions from CT and contour data. The proposed AttUNet model additionally leverages an attention mechanism to incorporate CT information. The predicted doses were transformed into deliverable plans using a dose mimicking approach (28). The resulting plans were validated on a 6MV linear accelerator with orthogonal stacked multileaf collimation. Testing on multi-institutional data affirmed the clinical applicability of the proposed models.

## 2 Materials and methods

### 2.1 Patient data

A total of 70 patients with solitary brain metastases treated with IMRT from April 2019 to July 2022 were recruited. Among them, 60 patients were selected from Radiotherapy Center 1, and the remaining 10 were selected from Radiotherapy Center 2. The training dataset consists of 36 patients randomly selected from Center 1. The subsequent evaluation of the models was conducted using the residual 34 patients from both centers.

The prescription dose for all patients was 30 Gy in 5 fractions. The dosimetric considerations for SRS were summarized as follows. The dose to 95% primary gross tumor volume (PGTV) reaches the prescription dose. Any areas receiving greater than 105% of the prescription dose, commonly referred to as high-dose spillage, are generally confined to the PGTV. For difficult cases, normal tissue volume receiving >105% of the prescription dose should be kept under 15% of the PGTV. Intermediate-dose spillage is responsible for most of the toxicity associated with SRS (29). The dose to any point 2 cm away from the PGTV surface (D2cm) should be below a limit. The ratio of 50% isodose volume (TV50%) to the PGTV volume (expressed as R50% = TV50%/PGTV) ought to be minimized to the greatest extent feasible.

### 2.2 Data processing

Using in-house Python scripts, the anatomical structure (RT-Struct), clinical dose distribution, and CT images were extracted from DICOM files. The coordinate systems of the structure and dose were aligned with the coordinate system of the CT image. Integer values denoted PGTV and organs at risk (OARs). Dose values were normalized to a range of -1 to 1. For enhanced image contrast, the CT values outside the range [-200, 300] were clipped to the interval edges. The resulting values were further normalized to [-1, 1]. To reduce computational complexity and save graphics memory, all data were cropped to 224 × 224 × 32 pixels with a pixel size of 2.5 × 2.5 × 2.5 mm. In the preliminary experiment, we found that the PGTV size greatly influences the model performance. As a result, we categorized training and test patients into two groups based on PGTV volume. Patients with a PGTV ≤ 20 cc were grouped under small PGTV, while those with a PGTV > 20 cc were labeled as large PGTV. In the training set, there were 36 cases in total: 15 cases in the large target area and 21 in the small target area. The test set comprised of 34 cases: 17 cases in the large target area and 17 in the small target area. Models were then trained and evaluated separately for each group.”

### 2.3 Neural network structure design and training

**Figure 1:**
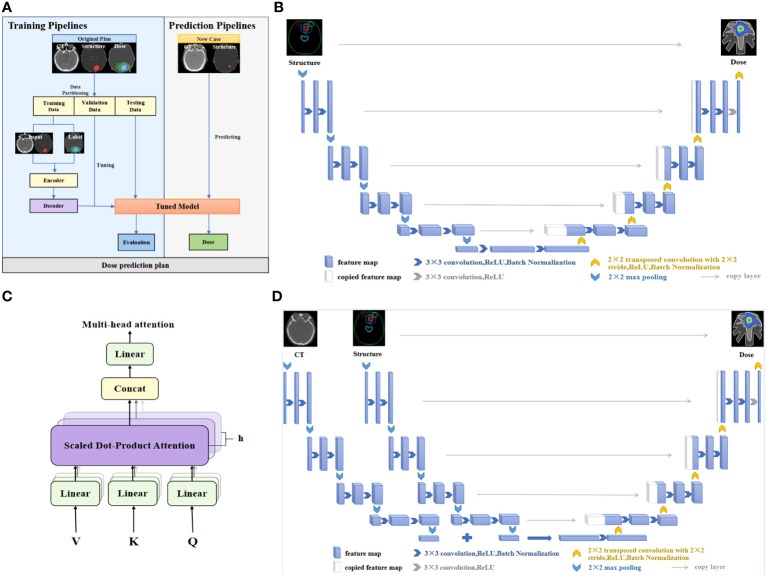
**(A)** Workflow of the dose prediction models. **(B)** UNet network. **(C)** Multi-head attention mechanism. **(D)** UNet equipped with multi-head attention (AttUNet). CT, computed tomography; ReLU, rectified linear unit.

We employed the deep learning framework PyTorch (Meta, Menlo Park, CA) to build dose prediction models. The first one is the 3D UNet which has been applied extensively to dose prediction tasks. The UNet model takes anatomical structures as input and outputs dose distribution. The second is our proposed 3D AttUNet, which uses anatomical structures and CT images as inputs. A novel attention mechanism was developed to facilitate the information fusion between CT images and anatomical structures. **Figure 1A** illustrates the two dose prediction models.

The widely used UNet network is designed to overcome the problem of resolution reduction caused by the pooling operation of convolutional neural networks. The network structure consists of two parts, namely, the encoder and the decoder. The encoder’s input layer is a 224×224×32 matrix, representing processed CT images and structural contours. The output consists of the extracted features of the anatomical structures. The decoder predicts a 224×224×32 dose matrix from the extracted features. As shown in **Figure 1B**, the encoder encompasses four layers bridged by 2x2x2 max pooling. Each layer incorporates dual 3x3x3 convolutions succeeded by batch normalization (BN) and rectified linear unit (ReLU) activation. The filter count commences at 32, and doubles post each pooling, peaking at 512 filters in the bottleneck layer. The decoder, mirroring the encoder, progressively upsamples the features to match the original input resolution. Skip connections amalgamate encoder and decoder features. UNet’s distinctive feature is its low-rank compact representation of anatomical structures, enhancing model generalizability.

The proposed AttUNet is an improved version of the above UNet. As illustrated in **Figure 1D**, two encoders extract features from the CTs and structures separately. The CT and structure features are fused by a multi-head attention layer before being passed to the decoder. The main purpose of attention is to allow an element in the structure features to look at all elements in CT features for clues that can help lead to a better encoding for this element. As illustrated in **Figure 1C**, we use 8 attention heads with 64 dimensional features per head. The attention layer introduces three matrices: Query, Key, and Value, to calculate the attention scores. The scores measure the importance of the key term compared to the query term related to a feature element. The key and value matrices are computed from the CT features with the dot product. The query matrix is obtained similarly from the structure features. The score is calculated by taking the dot product of the query with the key of the respective element we are scoring. The attention block is further refined by adding a mechanism called multi-head attention. It expands the model’s ability to focus on different positions and gives the attention layer multiple representation subspaces. The decoder, akin to UNet, comprises four upsampling layers.

We initialized the neural networks’ parameters with Kaiming initialization (30). The mean squared error between the inputs and outputs was used as the loss function. The network parameters were optimized on two NVIDIA 3090 GPUs using Adam optimizer with a learning rate set at 0.0001 and a batch size of 2. Whenever the validation loss plateaued, the learning rate was reduced by a factor of 10. Model training spanned 1000 epochs, incorporating an early stopping criterion if the validation loss saw no improvement over 100 consecutive epochs.

### 2.4 Dosimetric comparison between clinical and predicted doses

We employed specific dosimetric parameters (31) to juxtapose the predicted doses with clinical doses.

For PGTV, the dosimetric parameters encompass the doses covering 2%, 98%, and 95% of the target volume (D2%, D98%, and D95%), mean dose (Dmean), homogeneity index (HI), conformity index (CI), and intermediate-dose spillage (R50% and D2cm). The dosimetric parameters for OARs include the maximum point dose in brainstem volume (brainstem Dmax), the volume of brainstem receiving 23 Gy (brainstem V23), optic nerve Dmax, optic chiasm Dmax, lens Dmax, lens Dmean, eyeball Dmax, eyeball Dmean, and hypophysis Dmax.

The CI is defined in equation (1), indicating the ratio of the volume of the isodose shell that receives the prescription dose to the PGTV volume (32). VPGTV is the PGTV volume. VRX is the volume enclosed by the prescription isodose line. PGTVRX is the volume of PGTV enclosed by the prescription isodose line. The closer the value of the CI is to 1, the higher the conformity of the target volume.


(1) $$CI=\frac{{\left[PGTV_{RX}\right]}^{2}}{V_{PGTV}\times V_{RX}}$$

HI is defined in equation (2), indicating the difference between the maximum dose, the minimum dose, and the average dose in the target region (33). The dose distribution in the target region is considered to be homogeneous when the HI = 0. The larger the HI value, the poorer the homogeneity of dose distribution in the target region.


(2) $$HI=\frac{D_{2\%}-D_{98\%}}{D_{mean}}$$

R50% is defined in equation (3), representing ratio of 50% isodose volume V50%RX to the PGTV volume VPGTV. A larger R50% indicates a poor dose drop.


(3) $$R_{50\%}=\frac{V_{50\%RX}}{V_{PGTV}}$$

D2cm is defined as the maximum dose within 2 cm outside the PGTV. A larger D2cm indicates a poor dose drop.

The dosimetric parameter difference between predicted and clinical doses was patient-wisely calculated as their absolute difference |δD| and normalized to the prescription dose (30 Gy). The paired-sample t-test was used to assess the statistical significance of the difference mentioned above. A P-value > 0.05 indicates that the predicted dosimetric parameters do not deviate from the clinical ones, whereas a P-value< 0.05 implies the predicted plan is significantly different from the clinical plan.

The dose-point difference between clinical and predicted doses was computed point-wisely as $\text{δ}\left(\text{r},\text{r}\right)={\text{D}}_{\text{c}}\left(\text{r}\right)-{\text{D}}_{\text{p}}\left(\text{r}\right)$ , where r represents the point position. The mean ( ${\text{μ}}_{\text{δ}\left(\text{r},\text{r}\right)}$ ), standard deviation ( ${\text{σ}}_{\text{δ}\left(\text{r},\text{r}\right)}$ ), and mean absolute error (MAE) ( $\text{MAE}=\frac{1}{\text{n}}\sum_{\text{i}}^{\text{n}}|{\text{D}}_{\text{c}}\left(\text{r}\right)-{\text{D}}_{\text{p}}\left(\text{r}\right){|}_{\text{i}}$ ) of δ(r,r) were calculated to assess the accuracy of the predicted dose.

We used the dice similarity coefficient (DSC) to assess the 3D isodose accuracy, $\text{DSC}\left(\text{α},\text{b}\right)=\frac{2\left|\text{α}\cap \text{b}\right|}{\left|\text{α}\right|+\left|\text{b}\right|}$ , where α represents the isodose volume for the clinical plan and b is the isodose volume for the predicted outcome. We also used the 3D gamma passing rate at 3 mm/3% to evaluate the prediction accuracy (34–36).

For SRS plans, the dose gradient and the hot spot’s positioning are also of paramount importance. Hence, we extracted the dose profile along the cross-section’s axis to assess both the dose gradient and the location of the hot spot.

### 2.5 Plan delivery and dosimetric verification

**Figure 2:**
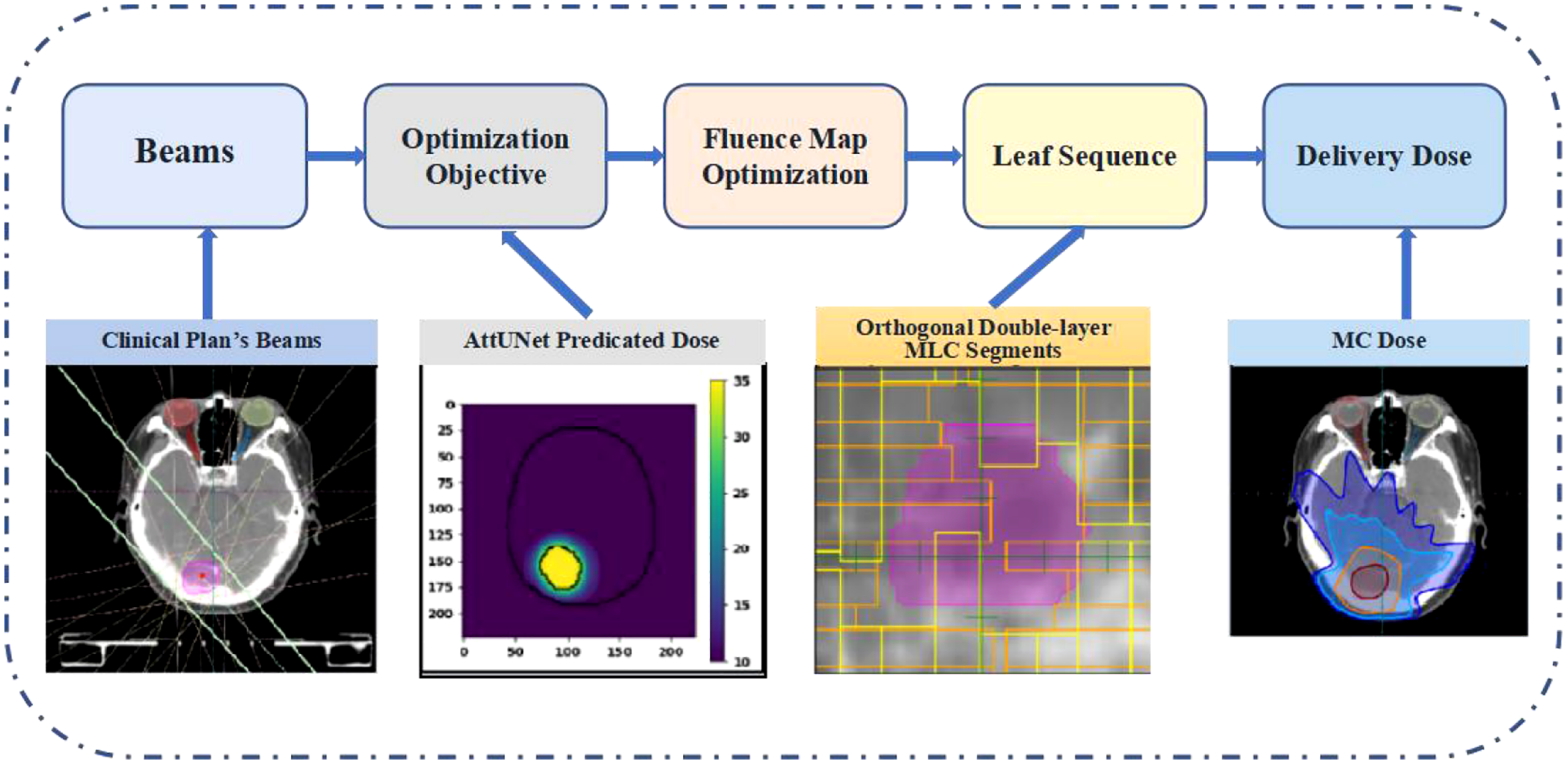
Pipeline of the dose mimicking. MLC, multi-leaf collimator; MC, Monte Carlo.

The predicted dose distributions were imported into LinaTech TiGRT TPS (LinaTech, Sunnyvale, CA) for dose mimicking. This approach enabled the creation of a clinically viable plan within the TPS system that mirrored the predicted dose without necessitating any alterations to the commercial software. Initially, coplanar beams were auto-configured based on institutional guidelines, eliminating the need for user intervention. Specifically, the inaugural beam direction is established by linking the center of the entire brain to the PTV’s center. Subsequently, four additional beams are introduced clockwise at 20-degree intervals from the primary beam. Another quartet of beams is added counter-clockwise, culminating in nine uniformly spaced beams. The optimizer then progressively refines the fluence intensity maps to curtail the voxel-wise disparity between the actualized and anticipated doses. The refined fluence maps are then transitioned into deliverable MLC sequences using the TPS’s inherent beam sequencing algorithms. A novel dual-layer orthogonal stacked MLC was used for the MLC sequencing, which collimates the 6 MV flattening-filter-free beam from a VenusX LINAC, as depicted in **Supplementary Figure 1**. The generated plans were then compared with clinical plans using the dosimetric parameters described in section 2.4. **Figure 2** provides the overview of the dose mimicking.

Based on our current hardware condition, the delivered doses were verified using the MatriXX Evolution 2D ionization chamber array and OmniPro IMRT (IBA Dosimetry, Schwarzenbruck, Germany) according to the routine for dose quality control. All the fields were set to gantry angle 0 and then exposed to the MatriXX to obtain the dose maps. The measured dose maps were then compared with the planned dose maps using the 2D gamma passing rate at 3 mm/3%.

## 3 Results

### 3.1 Dosimetry index statistics and DVH comparison

**Figure 3:**
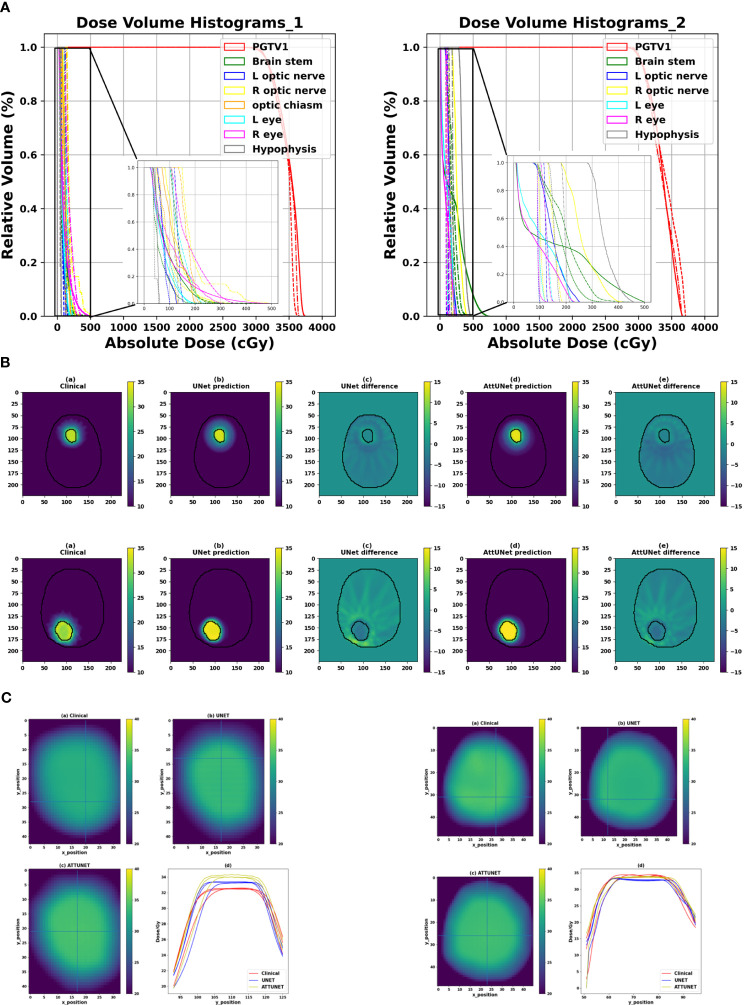
**(A)** Comparison of the DVHs of two test cases from two clinical centers (solid: clinical dose, dashed-dotted: prediction dose of UNet, dotted: prediction dose of AttUNet). **(B)** Illustrated dose distribution and the corresponding dose difference for the two patients ( $D_{DoseDifference}=D_{\mathrm{Pr}edictedDose}-D_{ClinicalDose}$ ). **(C)** Dose heatmaps of the two patients (top left: clinical dose, top right: UNet dose, bottom left: AttUNet dose, bottom right: normalized dose profile along the Y-axis of the cross lines). DVH, dose-volume histogram; PGTV, primary gross tumor volume.

**Figure 3A** shows the DVHs of the predicted and clinical plans for two test patients from two clinical centers. For both models, the DVHs of the predicted doses were close to the DVHs of the clinical plans. And the PGTV D95% of the predicted doses reached the prescription dose.

**Figure 3B** shows the dose distributions of the clinical plan, the predicted plan, and their absolute difference for the two test patients. We observed two models produced similar dose distributions and achieved a slightly better intermediate-dose spillage than the clinical plans.

**Figure 3C** shows that both models controlled the hot spot within the target region. The intersecting point of the cross lines indicates the maximum dose. We also plot the normalized dose profile along the vertical cross line in the bottom right subfigure. We found the high-dose spillage was confined within the PGTV area.

### 3.2 Dosimetric parameters of the predicted dose

**Table 1-1** presents the dosimetric values for the small PGTV group across the two models, while **Table 1-2** delineates the dosimetric values for the large PGTV group. It was observed that the doses produced by both UNet and AttUNet closely matched the clinical benchmarks in terms of D98%, D95%, D50%, D2%, Dmean, HI, and CI. With UNet, the statistical analysis indicated that six out of the seven dosimetric parameters in the small PGTV group significantly deviated from the clinical dose. In contrast, only one parameter in the large PGTV group demonstrated a statistically significant variation from the clinical dose. Conversely, the proposed AttUNet yielded statistically indistinguishable dose distributions across all seven parameters in the small PGTV group. Notably, even though the predicted D95% value by both models in the small PGTV group was statistically different from the clinical dose, the actual and predicted dose values were remarkably similar in each instance, with only negligible differences. Additionally, both UNet and AttUNet exhibited a steeper dose fall-off surrounding the PGTV in the large PGTV group compared to the clinical plan, as evidenced by the D2cm and R50% values. AttUNet manifested a more consistent dose gradient than UNet, as highlighted by the P-value.

**Table 1–1:** Target dosimetric parameters of small PGTV group.

	Dosimetric index	Clinical	UNet	$|\text{δDI}{|}_{\text{UNet}}$ (%)	P-value _UNet_	AttUNet	$|\text{δDI}{|}_{\text{AttUNet}}$ (%)	P-value_AttUNet_
PTV	D98%(Gy)	27.8 ± 0.9	27.4 ± 2.2	2.6 ± 6.2	0.31	28.0 ± 0.7	1.1 ± 1.3	0.19
	D95%(Gy)	28.7 ± 0.7	28.7 ± 0.7	0.2 ± 0.2	**0.00**	28.7 ± 0.7	0.2 ± 0.1	**0.00**
	D50%(Gy)	32.1 ± 0.7	33.7 ± 1.0	5.4 ± 3.5	**0.00**	32.4 ± 0.9	3.0 ± 2.3	0.26
	D2%(Gy)	34.3 ± 1.4	36.7 ± 1.5	8.3 ± 6.3	**0.00**	34.6 ± 0.8	4.9 ± 4.0	0.52
	Dmean(Gy)	31.9 ± 0.7	33.2 ± 0.9	4.7 ± 3.2	**0.00**	32.1 ± 0.8	2.7 ± 2.1	0.33
	HI	0.20 ± 0.06	0.28 ± 0.09		**0.00**	0.20 ± 0.02		0.83
	CI	0.79 ± 0.04	0.84 ± 0.04		**0.00**	0.81 ± 0.08		0.38
Body	D2cm(Gy)	13.2 ± 2.5	15.2 ± 3.5	18.6 ± 7.0	**0.01**	15.3 ± 2.4	18.5 ± 8.4	**0.01**
	R50	2.7 ± 0.3	2.4 ± 0.3		**0.02**	2.9 ± 0.4		**0.02**

The values were calculated over 34 patients and reported in Mean (± SD) format. Bold fonts indicate statistical significance.

**Table 1–2:** Target dosimetric parameters of large PGTV group.

	Dosimetric index	Clinical	UNet	$|\text{δDI}{|}_{\text{UNet}}$ (%)	P-value _UNet_	AttUNet	$|\text{δDI}{|}_{\text{AttUNet}}$ (%)	P-value_AttUNet_
PTV	D98%(Gy)	28.3 ± 1.2	28.0 ± 1.5	3.7 ± 5.7	0.54	27.5 ± 1.2	4.8 ± 4.6	0.14
	D95%(Gy)	29.4 ± 0.4	29.4 ± 0.4	0.2 ± 0.2	0.05	29.4 ± 0.6	0.5 ± 0.9	0.99
	D50%(Gy)	32.9 ± 1.4	34.2 ± 2.9	7.7 ± 10.0	0.19	34.7 ± 4.1	9.4 ± 13.5	0.15
	D2%(Gy)	35.0 ± 2.4	35.9 ± 3.2	11.1 ± 10.9	0.50	36.3 ± 4.2	12.7 ± 13.2	0.38
	Dmean(Gy)	32.6 ± 1.3	33.5 ± 2.6	6.9 ± 8.7	0.32	33.9 ± 3.5	8.3 ± 11.4	0.22
	HI	0.20 ± 0.09	0.22 ± 0.10		0.60	0.24 ± 0.10		0.33
	CI	0.85 ± 0.04	0.87 ± 0.04		**0.02**	0.86 ± 0.05		0.28
Body	D2cm(Gy)	17.4 ± 5.6	12.9 ± 2.9	36.6 ± 16.4	**0.00**	13.2 ± 3.6	36.1 ± 16.1	**0.00**
	R50	2.2 ± 0.2	1.8 ± 0.3		**0.00**	1.8 ± 0.4		**0.00**

The values were calculated over 34 patients and reported in Mean ± SD format. Bold fonts indicate statistical significance.

**Table 2** summarizes the dosimetric parameters of OARs for the two models. For 11 out of 14 patients, the difference in dosimetric parameters between predicted and clinical doses were not statistically significant. For the three deviating parameters (left, right eye Dmean, and right eye Dmax), UNet achieved lower toxicity AttUNet, although the dose distribution produced by AttUNet did not exceed the dose limitation of the eye.

**Table 2:** OARs dosimetric parameters.

	Dosimetric Parameters	Clinical	UNet	$|\text{δDI}{|}_{\text{UNet}}$ (%)	*P*-value UNet	AttUNet	$|\text{δDI}{|}_{\text{AttUNet}}$ (%)	*P*-value AttUNet
Brain Stem	Dmax (Gy)	9.1 ± 6.9	9.3 ± 9.1	9.6 ± 7.6	0.77	10.2 ± 9.3	10.6 ± 8.6	0.20
	V23 (%)	0.1 ± 0.2	0.1 ± 0.4	0.2 ± 0.8	0.14	0.1 ± 0.4	0.2 ± 0.7	0.15
Optic Nerve L	Dmax (Gy)	2.3 ± 3.7	2.0 ± 3.5	2.4 ± 3.4	0.31	2.5 ± 3.9	3.1 ± 3.2	0.41
Optic Nerve R	Dmax (Gy)	2.1 ± 2.4	1.5 ± 0.9	3.9 ± 4.8	0.11	2.1 ± 1.1	4.2 ± 4.4	0.92
Optic Chiasm	Dmax (Gy)	2.8 ± 2.3	2.1 ± 1.6	4.9 ± 4.6	0.17	2.9 ± 1.7	5.3 ± 5.1	0.80
Len L	Dmax (Gy)	0.4 ± 0.3	0.9 ± 0.4	1.9 ± 1.4	0.20	1.2 ± 0.2	2.6 ± 4.3	0.09
	Dmean (Gy)	0.3 ± 0.2	0.8 ± 0.4	1.8 ± 1.1	0.19	1.1 ± 0.2	2.6 ± 0.3	0.08
Len R	Dmax (Gy)	0.6 ± 0.6	1.0 ± 0.5	1.5 ± 1.3	0.19	1.3 ± 0.4	2.4 ± 0.5	0.09
	Dmean (Gy)	0.4 ± 0.3	0.9 ± 0.5	1.9 ± 1.3	0.16	1.2 ± 0.4	2.9 ± 0.4	0.09
Eye L	Dmax (Gy)	1.5 ± 1.6	1.2 ± 0.8	2.1 ± 2.2	0.12	1.8 ± 1.0	2.7 ± 1.8	0.14
	Dmean (Gy)	0.7 ± 0.7	0.8 ± 0.4	1.2 ± 0.9	0.13	1.4 ± 0.6	2.5 ± 1.5	**0.00**
Eye R	Dmax (Gy)	1.8 ± 1.4	1.3 ± 0.7	3.0 ± 2.3	**0.04**	1.9 ± 1.0	2.7 ± 2.2	0.54
	Dmean (Gy)	0.8 ± 0.6	0.9 ± 0.5	1.3 ± 0.8	0.22	1.5 ± 0.7	2.4 ± 1.6	**0.00**
Hypophysis	Dmax (Gy)	2.1 ± 2.0	1.5 ± 0.9	3.9 ± 3.0	0.11	2.3 ± 1.0	4.8 ± 3.4	0.67

PGTV, primary gross tumor volume; HI, homogeneity index; CI, conformity index; D2%, D98%, and D95%, minimum dose to 2%; 98%; and 95% of the target volume; respectively; D_2cm_, maximum dose within 2 cm outside the PGTV; DI, dose-related index; Dmean, mean point dose in the target volume; Dmax, maximum point dose in the target volume; OAR, organ at risk. The values were calculated over 34 patients and reported in Mean ( ± SD) format. Bold fonts indicate statistical significance.

In summary, **Tables 1-1**, **1-2**, and **2** suggest that the two models produced clinically acceptable dose predictions, and AttUNet outperformed UNet slightly.

### 3.3 Comparison of the two prediction models

**Figure 4:**
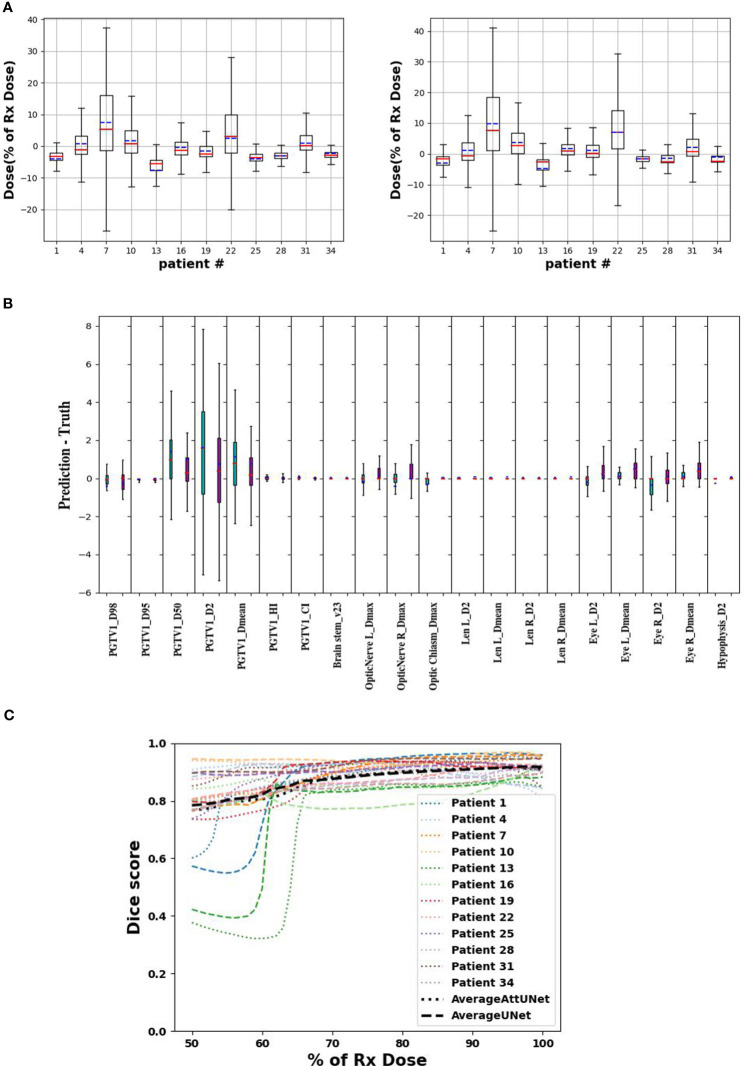
**(A)** point-wise dose difference between predicted and clinical doses for UNet (left) and AttUNet (right). Mean, median, and standard deviation are plotted in blue, red, and box, respectively. **(B)** patient-wise dosimetric difference of UNet (green) and AttUNet (purple). **(C)** dice score of the two models as a function of relative dose for 12 test patients. PGTV, primary gross tumor volume; OARs, organs at risk.

Dose difference between clinical and predicted distributions was computed point-wisely. **Figure 4A** plots the median, mean, and standard deviation of the dose difference for 12 test patients. The difference was rescaled relative to the prescription dose. The two models showed similar predictive ability among the test patients. For example, they both predicted large dose differences for patient 7 and 22. And they both achieved small dose differences for patient 1, 25, 28, and 34. Overall, AttUNet achieved a smaller average MAE (4.3%) for all patients than UNet (4.4%). The maximum MAE was 13.8% for UNet and 10.6% for AttUNet. These findings indicate that AttUNet performed more closely in line with the desired outcomes than UNet when assessing point-wise dose difference.

**Figure 4B** compares the dosimetric parameter difference of UNet and AttUNet. For 16 out of 20 parameters, AttUNet surpassed the UNet.

**Figure 4C** compares the DSC of clinical and predicted doses for 12 test patients. The DSC of 1 indicates an ideal match of 3D isodose surface distribution. The predicted doses were close to the clinical doses in the high-dose area, while the model performance in the low-dose area was relatively poor.

**Figure 5:**
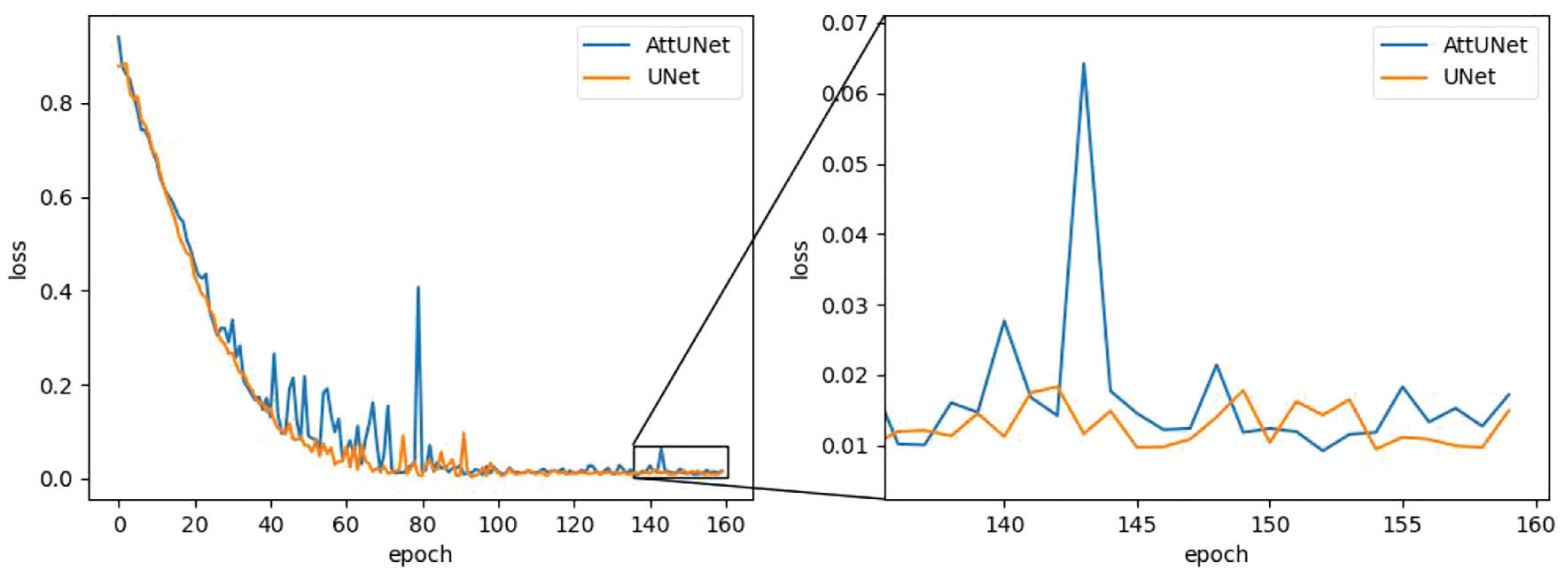
Training loss as a function of the training epoch for the two models. MSE, mean square error.

While AttUNet generally outperformed UNet, it is harder to train, as illustrated in **Figure 5**, where training loss (weight MSE) was plotted as the function of the training epoch. We found the training dynamic of UNet is quite stable, while AttUNet fluctuated over the training process.

### 3.4 Dose delivery verification

Two deliverable plans were generated from the predicted doses following the dose-mimicking pipeline shown in **Figure 2**. Dose mimicking is achieved by optimizing fluence maps to replicate the predicted dose distribution closely. Typically, this optimization spans between 1 and 5 minutes. Following this, the fluence maps are seamlessly transformed into MLC sequences, upon which a Monte Carlo dose algorithm calculates the ultimate delivery dose. This phase necessitates a duration of roughly 2 to 10 minutes.

**Figure 6:**
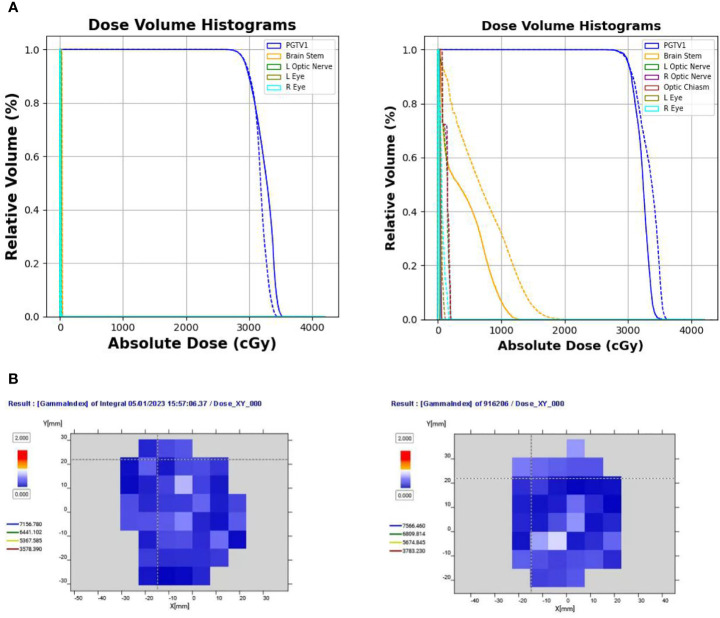
**(A)** Comparison of DVHs of two test cases from two clinical centers (solid line indicates the clinical dose; dotted line indicates the delivery dose). **(B)** Gamma pass rate (%) of the two delivered plans under the 3 mm/3% criterion. DVH, dose-volume histogram.

Their DVHs, gamma passing rates, and dosimetric parameters are shown in **Figures 6A**, **6B**, and **Table 3**, respectively.

**Table 3:** Dosemetric parameters of the delivered plans.

	Dosimetric Parameters	Patient 1		Patient 2	
		Clinical	Delivery	Clinical	Delivery
PGTV	D98% (Gy)	27.6	27.3	28.4	28.5
	D95% (Gy)	29.4	29.5	29.2	29.2
	D50% (Gy)	35.9	34.1	32.1	33.1
	D2% (Gy)	39.8	36.9	33.8	34.6
	Dmean (Gy)	35.3	33.7	31.8	32.6
	HI	0.34	0.28	0.17	0.18
	CI	0.78	0.78	0.86	0.88
Body	D2cm (Gy)	15.4	16.6	14.1	13.2
	R50	3.5	3.5	2.3	2.4
Brain Stem	Dmax (Gy)	10.0	11.2	13.3	19.5
	V23 (%)	0.0	0.0	0.0	0.0
Optic Nerve L	Dmax (Gy)	3.4	3.4	0.3	1.1
Optic Nerve R	Dmax (Gy)	10.2	7.0	0.4	1.2
Optic Chiasm	Dmax (Gy)	7.1	6.9	0.5	1.2
Len L	Dmax (Gy)	0.7	0.6	0.0	0.0
	Dmean (Gy)	0.6	0.4	0.0	0.0
Len R	Dmax (Gy)	1.4	2.9	0.0	0.0
	Dmean (Gy)	0.8	2.6	0.0	0.0
Eye L	Dmax (Gy)	3.1	2.0	0.2	1.1
	Dmean (Gy)	1.8	0.9	0.2	1.0
Eye R	Dmax (Gy)	4.8	4.3	0.3	1.1
	Dmean (Gy)	2.7	2.8	0.2	1.0
Hypophysis	Dmax(Gy)	6.6	7.2	0.4	1.2

**Figure 6A** compares the DVHs of the delivered plans and clinical plans. **Table 2** reports the PGTV and OAR dosimetric parameters of the delivered plans and clinical plans. The results suggest that the delivered plans met the clinical criteria.

**Figure 6B** shows the gamma pass rate of the two delivered plans at the 3 mm/3% criterion. Both plans achieved a 100% passing rate, indicating successful delivery.

### 3.5 Automatic planned quality and efficiency assessment

**Figure 7:**
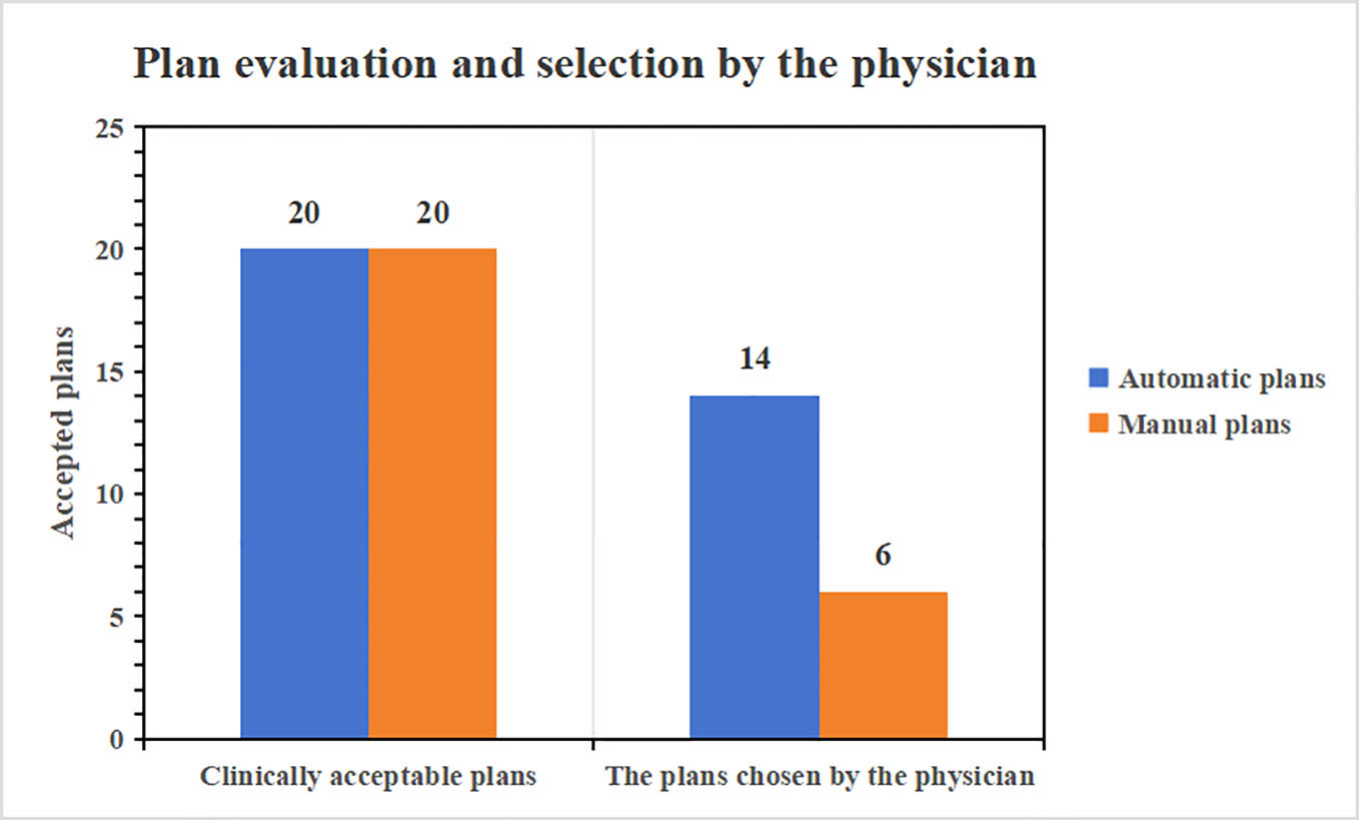
Plan assessment conducted by a physician with 10 years of experience.

The 20 auto-generated plans underwent a thorough reevaluation by a physician boasting a decade of planning expertise, confirming that all were clinically acceptable. Furthermore, when the automatic plans were juxtaposed with their corresponding manual versions for the 20 cases and presented to the physician for assessment, they were tasked with selecting the superior plan. Impressively, 14 of the auto-generated plans were the favored choice of the radiologist. Given that they fulfill clinical prerequisites, the automatic plans exhibit swifter fall-down in low doses and superior low-dose protection compared to their manual counterparts. **Figure 7** provides a comprehensive visual representation of the clinical pass rate and the physician’s selection criteria.

**Supplementary Table 1** enumerates the optimization durations for both automatic and manual plans. Specifically, for solitary brain metastase plans, the mean and standard deviation of optimization time are tabulated as ([481.2 ± 55/1212 ± 237.7] s, [Automatic/manual]). In this study, the fluence map’s automated generation utilized a deposition matrix, a method proposed by M.C. The strategy ensures target coverage and observes organ-at-risk constraints. It also considers the dose gradient beyond the target region within the stereotactic radiotherapy plans’ context. This requires the harnessing of the patient’s entire CT voxel dataset. Consequently, this method processes a more substantial voxel dataset than the automatic plans designed for conventional fractionated doses, which might account for a more extended duration. However, creating automated plans that adhere to clinical standards undeniably offers a time-saving advantage over manual plan formulations.

## 4 Discussion

Two deep-learning dose prediction models based on 3D UNet have been developed and evaluated for brain metastases SRS. The comparison of the predicted and clinical doses shows that the average MAE was 4.4% and 4.3% for UNet and AttUNet, respectively. The dice similarity coefficient of isodose volume above 65% of the prescribed dose exceeded 85%. The predicted doses had a sharp dose gradient, and hot spots fell within the target region. The dose verification results show that the deliverable plans achieved a 100% gamma pass rate at 3 mm/3% and met the dose evaluation criteria. We also observed some unfavorable issues. For instance, in the low-dose area (below 65% of the prescribed dose), the model prediction results varied considerably from the clinical plan. For some patients, the DSC of isodose was less than 85%, suggesting the models’ heightened focus on PGTV. Training loss metrics highlighted that AttUNet experienced more fluctuating training dynamics compared to UNet’s steady convergence. This inconsistency could be attributed to several factors:

The integrated attention mechanism amplifies the model’s complexity, complicating optimization.

AttUNet grapples with assimilating information from two diverse data sources (CTs and structures), whereas UNet exclusively processes structures. Streamlining this fusion is challenging.

The restricted dataset size might be inadequate for effectively training the augmented parameters in AttUNet. Potential solutions encompass employing advanced optimization strategies, enhancing data augmentation, modifying architectural components, and rigorously monitoring validation performance. Further research is paramount to facilitate the robust and efficient training of intricate multimodal structures like AttUNet for dose prediction endeavors.

While deep learning has been rigorously explored for dose prediction in radiotherapy planning, making direct comparisons with extant models remains challenging due to differences in treatment modalities and patient datasets used across studies. Campbell (37) noted an MAE discrepancy for SBRT for pancreatic cancer not surpassing 10%, while our AttUNet model registered a peak MAE of 10.6%. Liu (38) disclosed a 3D gamma pass rate of 81.5-93.4% for helical tomotherapy for nasopharyngeal carcinoma utilizing a U-ResNet-D model. Our study’s corresponding figures were 87.9% for UNet and 85.5% for AttUNet at 3 mm/3% for brain metastases.

A prevalent limitation in contemporary dose prediction techniques is the omission of deliverable plan verification. The predicted dose distributions are not necessarily clinically deliverable, even though they are derived from previously deliverable treatment plans. The current work, therefore, completed the entire automated planning pipeline in a closed-loop framework. Furthermore, the proposed AttUNet incorporates the multi-head attention mechanism to combine features from CT images and anatomical structures. This integration aims to discern both commonalities and differences, potentially enhancing the model’s efficiency. Our experiments, based on this specific dataset, indicated that AttUNet’s performance was more in line with the desired outcomes than UNet across various criteria. However, it’s important to note that these findings might not be generalizable to other datasets given the limited scope of our testing. Tests across two centers validated the model’s predictive prowess in a multi-center setting. Utilizing the forecasted dose as an initial reference for planning assures consistent plan quality, independent of planner, TPS, and LINAC variables. The automated planning method proposed herein operates autonomously, devoid of manual intervention, culminating in 3-17 minutes. In contrast, traditional planning durations for multi-met SRS cases using HyperArc have been documented at 77 minutes (39). Thus, our novel methodology significantly curtails planning durations by obviating labor-intensive manual iterations.

While the models we have developed exhibit encouraging outcomes for single-target brain metastases SRS, several constraints warrant attention. Firstly, these models have been tailored and assessed exclusively for isolated brain metastases, a scenario markedly simpler than multi-lesion occurrences. Adapting these models to cater to multiple lesions may necessitate alterations to accommodate the interplay among adjacent abnormalities. In our investigations, mitigating the toxicities in surrounding healthy tissue proved straightforward, mainly because the organs at risk (OARs) were distinctly separated from the targeted region. Predicting doses for instances where OARs are juxtaposed or even encroach upon the targets could intensify the challenge. Thirdly, our models have been calibrated for a singular prescription dose tier, leaving their adaptability to fluctuating prescription doses uncharted. Fourthly, while our models forecast a solitary dose distribution, clinical protocols might see multiple potential dose distributions for a single patient. Variabilities might stem from institutional preferences, patient-centric considerations, or LINAC specifications. Peering into the future, enhancing the models might involve training on diversified datasets encompassing multiple metastases and an array of prescriptions. Introducing sophisticated network structures could amplify dose conformality and organ conservation. Estimating uncertainties could pinpoint scenarios where model predictions might waver. A culmination of biological modeling with radiomic data might pivot predictions towards clinical outcomes, transcending mere physical dose distributions. In essence, this research paves the way for automation in planning, yet a concerted effort is essential to navigate intricate clinical landscapes.

Our study developed and evaluated an automatic brain-metastases-SRS planning pipeline using deep learning for dose prediction. While automated planning techniques have been explored for Gamma Knife radiosurgery, this work represents a novel application of deep learning to predict dose distributions specifically for stereotactic radiosurgery of brain metastases delivered by a linear accelerator platform with multi-leaf collimator beam modulation, as well as verification of the deliverable treatment plans. Both models yielded clinically acceptable predicted doses in our study. The prediction outcomes from AttUNet were more consistent with the original clinical plan compared to those from UNet. The predicted dose distribution of the two models fulfilled the clinical prerequisites in terms of target region dose, dose drop, and protection of organs at risk. The deliverable plans agreed with the predicted doses regarding the gamma passing rate and achieved clinical criteria. When applied to other treatment sites or modalities, the proposed models may require adaptation. The 3D dose projections proffered by our models stand to refine radiotherapy planning blueprints, ensuring consistent plan quality and underscoring the potential for a fully automated radiotherapy treatment planning paradigm.

## Data availability statement

The raw data supporting the conclusions of this article will be made available by the authors, without undue reservation.

## Author contributions

JP: Conceptualization, Data curation, Formal analysis, Investigation, Methodology, Resources, Software, Validation, Writing – original draft, Writing – review & editing. JX: Data curation, Methodology, Software, Writing – review & editing. CR: Methodology, Validation, Writing – review & editing. QS: Methodology, Writing – review & editing. LS: Funding acquisition, Project administration, Validation, Writing – review & editing. FZ: Formal analysis, Investigation, Resources, Supervision, Writing – review & editing. HJ: Conceptualization, Data curation, Formal analysis, Software, Writing – review & editing. XL: Data curation, Funding acquisition, Project administration, Supervision, Validation, Visualization, Writing – original draft, Writing – review & editing.
