# Supplementary material for: Deep-learning-driven dose prediction and verification for stereotactic radiosurgical treatment of isolated brain metastases
Source: Front Oncol. 2023 Nov 20;13:1285555. doi: 10.3389/fonc.2023.1285555 (PMC10701271; doi:10.3389/fonc.2023.1285555)
Supplement: Supplementary file 1 [file DataSheet_1.docx]

Supplementary Material

# Supplementary Figures and Tables

## Supplementary Figures


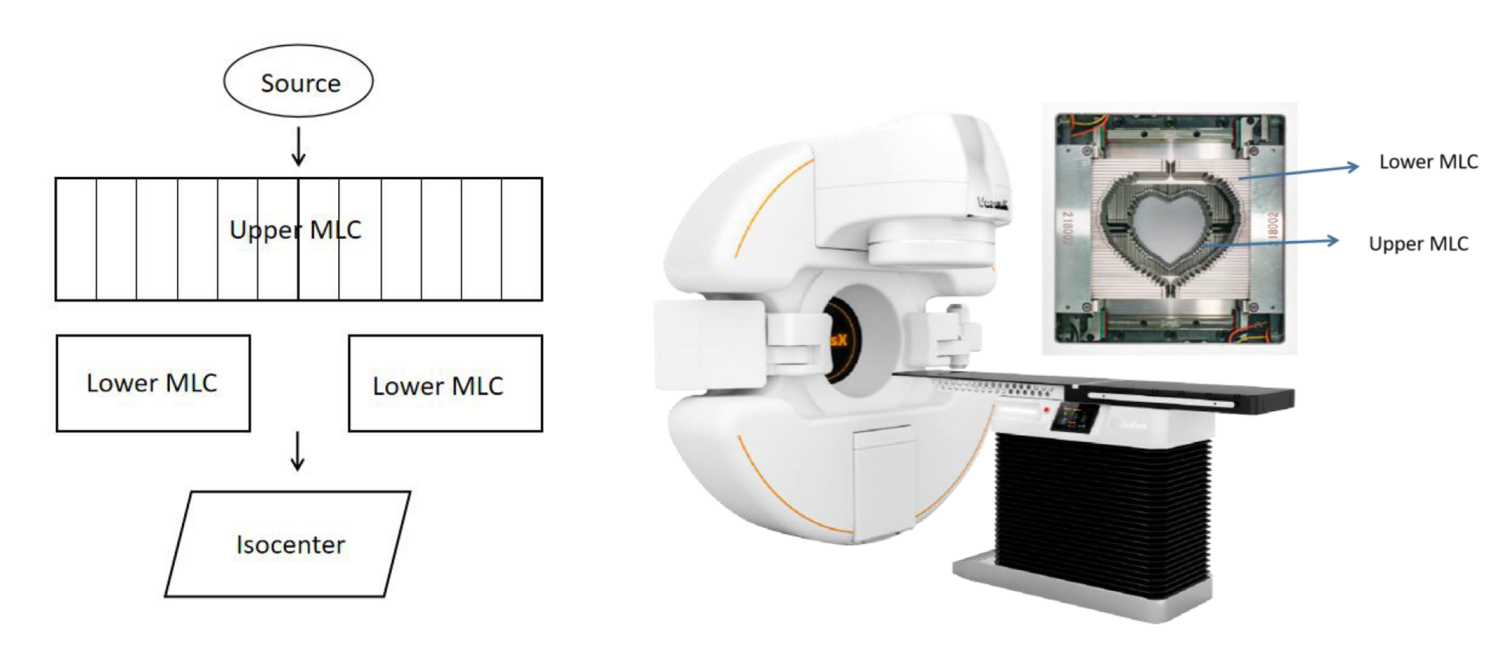


**Supplementary Figure 1.** Schematic diagram of orthogonal dual-layer multi-leaf collimator αMLC. **(A)** Simplified schematic diagram of the αMLC installation configuration; **(B)** physical diagram of the αMLC.

## Supplementary Figures

**Supplementary Table 1**. Optimization time statistics for Automatic plans and manual plans

|  |  | Dose prediction (s) | Generating fluence map (s) | Generate control points and dose calculations (s) | Total time (s) |
| --- | --- | --- | --- | --- | --- |
| Plans | Automatic plans | 1.2±0.1 | 121.6±24.3 | 358.4±30.6 | 481.2±55 |
|  | Manual plans | / | | | 1212±237.7 |
